# Case report: Early thrombosis in left atrial during transcatheter closure of ASD in a child with favorable outcome after use of GPIIb/IIIa receptor antagonist and heparin

**DOI:** 10.3389/fped.2023.1138717

**Published:** 2023-03-13

**Authors:** Hui Wang, Zhiwei Zhu, Zhenyu Liu, Yonghua Yuan, Xuan Xu, Liping Liu, Jie Wen, Xiaohui Xia, Yu Zhang, Jin He

**Affiliations:** ^1^ Department of Pediatric Cardiology, Children’s Medical Center, Hunan Provincial People's Hospital, The First Affiliated Hospital of Hunan Normal University, Changsha, China; ^2^ Department of Pediatric Orthopedics, Hunan Provincial People's Hospital, The First Affiliated Hospital of Hunan Normal University, Changsha, China; ^3^ Department of Ultrasound, Hunan Provincial People's Hospital, The First Affiliated Hospital of Hunan Normal University, Changsha, China; ^4^ Department of Cardiology, Children’s Medical Center, Hunan Provincial People's Hospital, The First Affiliated Hospital of Hunan Normal University, Changsha, China

**Keywords:** ASD, thrombosis, complications, tirofiban, children, case report, catheterization

## Abstract

**Background:**

Acute thrombus in atrial septal defect occluders is a rare complication that requires aggressive, effective, and safe management. Tirofiban, a platelet glycoprotein IIb/IIIa receptor antagonist, is widely used for the management of thromboembolic diseases, such as coronary heart disease and stroke. To date, there is no report using the GPIIb/IIIa receptor antagonist tirofiban for the management of ASD closure-related thrombosis in children.

**Case presentation:**

Herein, we reported a case of a 5-year-old girl with ASD who presented with acute thrombus on the left disc of the occluder device immediately after transcatheter closure of ASD. The thrombus was successfully dissolved 24 h after a combined infusion of heparin and tirofiban, followed by 1 months of aspirin and clopidogrel and 5 months of aspirin alone. No thromboembolism or hemorrhage events occurred during follow-up for more than 2 years.

**Conclusion:**

The continuous infusion of GPIIb/IIIa receptor antagonist tirofiban combined with heparin may have beneficial effects for the management of thrombosis during ASD closure procedure.

## Introduction

Atrial septal defect (ASD) is one of the most common congenital heart diseases (CHD), accounting for approximately 6%–10% of the CHD population (1, 2). Over the years, percutaneous transcatheter closure of ASD has replaced the traditional thoracotomy, becoming the preferred treatment for secondary ASD (3–7). Since King TD first performed transcatheter closure of ASD in 1976 (3), there has been rapid development of materials and equipment used for transcatheter closure techniques. The accessibility, hospitalization and complications have been largely improved. However, infrequent adverse events can still occur related to ASD closure, such as movement or embolization of the closure device, pericardial effusion, arrhythmias, thrombosis, mitral insufficiency and vascular injury (8–10).

Thrombus formation during closure of ASD is a rare but severe complication. Tirofiban, a platelet glycoprotein IIb/IIIa receptor antagonist, is widely used for the management of thromboembolic diseases, such as coronary heart disease and stroke. Nonetheless, tirofiban is rarely administrated for ASD closure-related thrombosis, especially for children. Here we report a single case of acute thrombosis during ASD closure with favorable outcome, treated with combination of tirofiban and heparin.

## Case presentation

A 5-year-old girl was admitted to our hospital on 17 August 2020 with a 3-year history of a heart murmur. She had no history of other cardiovascular, hematologic, or neurological diseases and no medication use.

The child was developing normally, without shortness of breath or cyanosis. Fixed splitting of the second heart sound and 2/6 systolic blowing murmur could be heard in the second intercostal space on the left sternal border. Blood examination showed the following: WBC 9.64 (×10^9^/L), N 4.45 (×10^9^/L), L 4.58 (×10^9^/L), Hb 136 (g/L), Plt 393 (×10^9^/L), and CRP <0.499. Biochemistry tests showed no abnormalities. Coagulation function were: TT:10.1 s, INR:0.90, FIB:3.00 g/L, APTT:25.4 s, TT:17.2 s, D-Dimer:0.45 mg/L, FDP:6.12 ug/ml, AT-IIIAg:92.7%, showing no abnormalities.

Preoperative preparation was completed. Intravenous general anesthesia was performed, and intravenous heparin anticoagulation was 100 u/kg. The intraoperative examination showed Qp : Qs ratio of 1.5 : 1 and pulmonary arterial pressure of 30 mmHg. Intraoperative transthoracic echocardiography (TTE) showed central secundum ASD (II) with a defect diameter of 8 mm, and the marginal conditions were suitable for closure.

**Figure 1:**
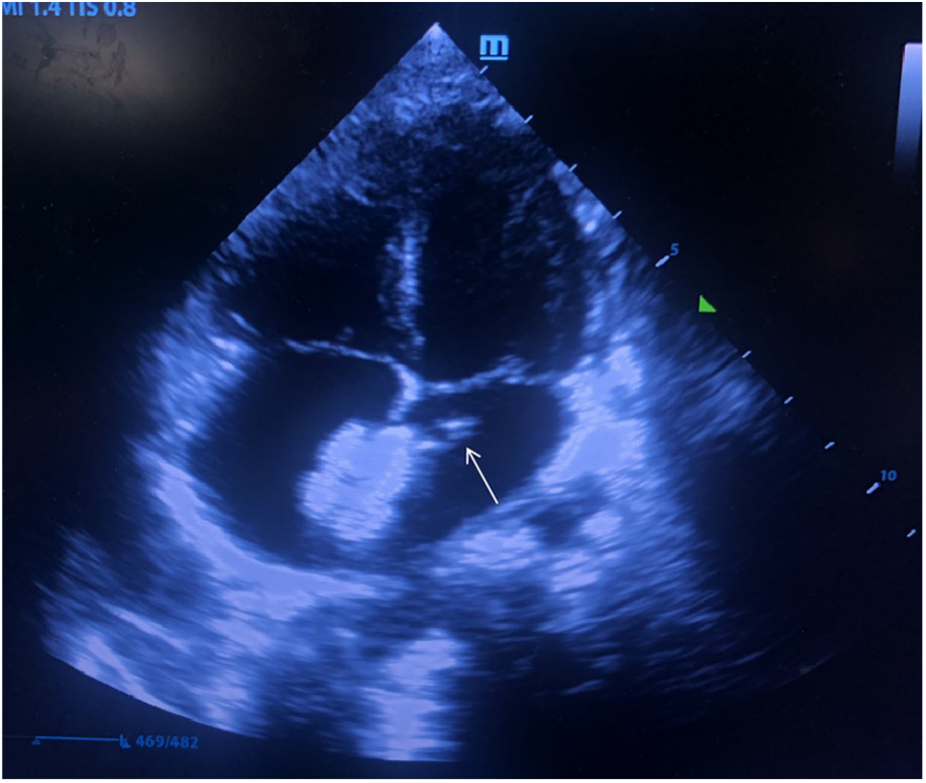
Floating thrombus in the edge of the left atrial disc of the occluder (white arrow).

A 12 mm Amplatzer atrial septal occluder (Starway Medical Technology, Inc. Beijing, China) was initially deployed in the atrial septum within 40 min after femoral vein puncture. Due to the unsatisfactory shape of the occluder by the first release, the left disc of the occluder was recollected into the delivery sheath and was released again. The procedure time from the recollection to the repositioning of occluder was estimated 15 min. After a second complete release of the occluder and withdrawal of the delivery sheath, the bedside TTE revealed a slightly hyperechoic mass (approximately 6.5 × 3 mm in size) attached to the edge of the left atrial disc of the deployed occluder, oscillating with the movement of cardiac cycle (Figure 1). An acute thrombosis was swiftly considered by the ultrasonographer and cardiologists.

**Figure 2:**
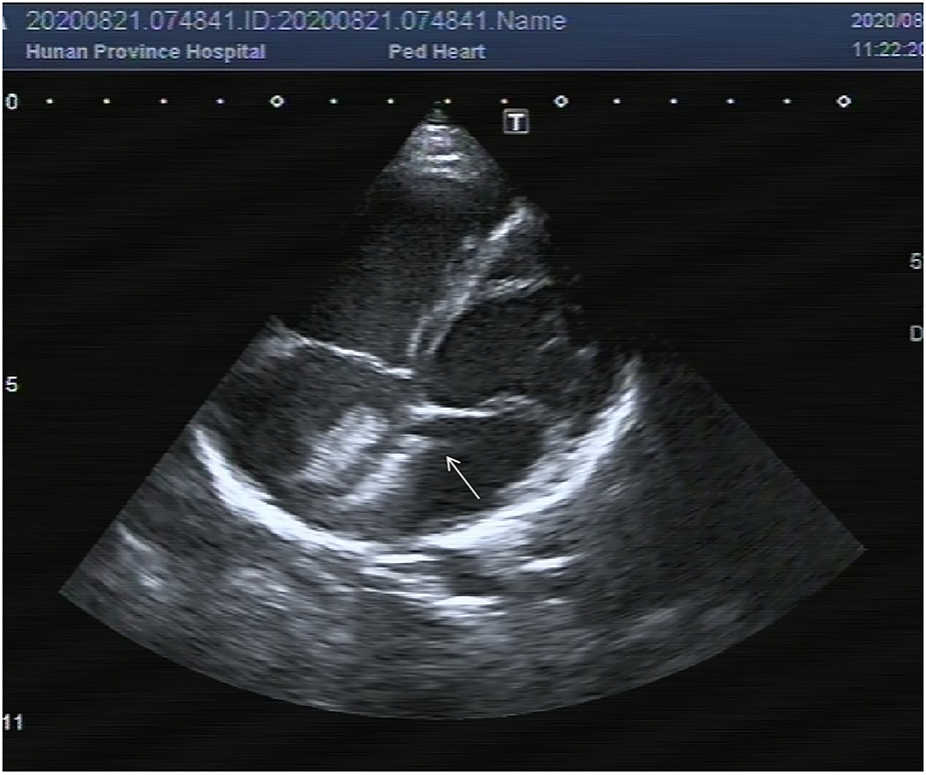
Thrombus disappeared over 90% 24 h after tirofiban and heparin infusion (white arrow).

After obtaining the informed consent, platelet glycoprotein IIb/IIIa receptor antagonist tirofiban 0.4 ug/kg/min was pumped immediately lasting for 30 min. Then 0.10 ug/kg/min tirofiban was used for continuous pumping for 24 h. After 24 h, TTE showed that the thrombus had disappeared, and the absorption rate was over 90% (Figure 2). During the infusion, the child had transient mild headache and chest tightness, lasting for about 3 h. No other discomfort or adverse events occurred, such as vomiting, bleeding or oozing at the puncture site. Since the headache and chest tightness are both common symptoms after general anesthesia, no particular intervention was given and the symptoms resolved without recurrence in the follow up.

Re-examination of coagulation function showed the following: blood routine showed WBC 8.29 (×10^9^/L), Hb 126 (g/L), and Plt 294 (×10^9^/L). Postoperative re-examination of coagulation function: PT: 10.7 s, INR: 0.93, FIB: 1.9 g/L, APTT: 26.8 s, TT: 17.2 s, D-Dimer: 0.89 mg/L, FDP: 6.30 ug/ml, AT-III Ag: 105.3%.

**Figure 3:**
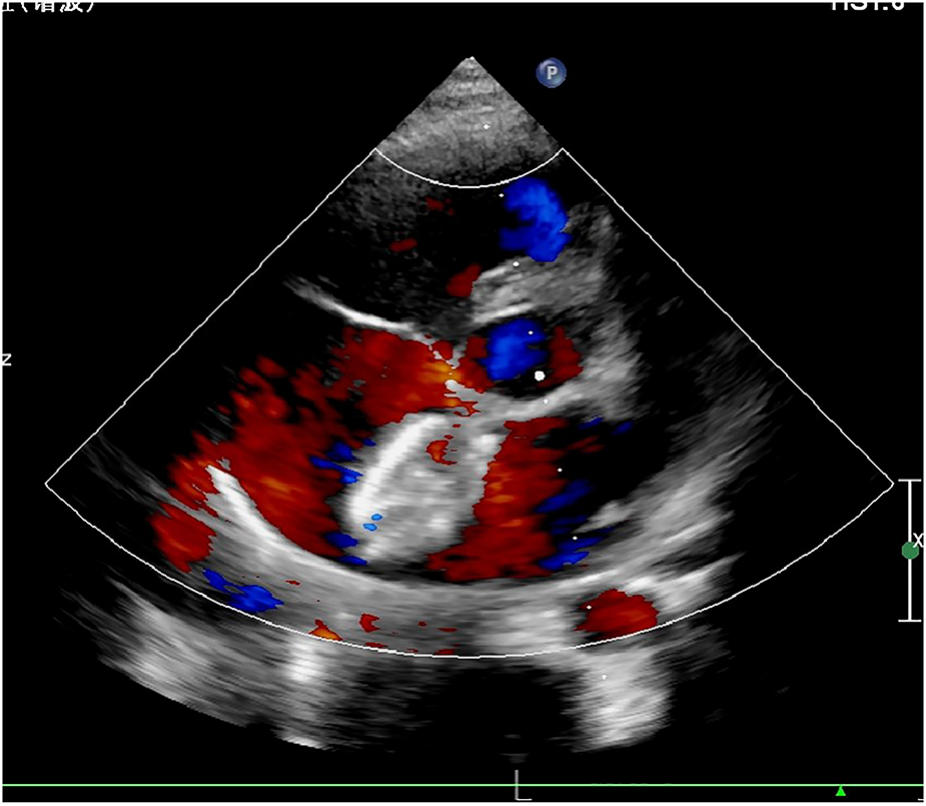
Transthoracic echocardiography at 1-month post-procedure showed that the thrombus was completely absorbed.

Tirofiban was discontinued 24 h later and replaced by aspirin and clopidogrel for anticoagulation. One month later, TTE showed that the occluder was in a good position, and the thrombus on the left disc of the occluder was completely dissolved (Figure 3). Aspirin alone was continued in the subsequent 5 months. TTE was then performed again at 3 months, 6 months, 1 year, and 2 years after surgery showing no recurrence of thrombus formation or bleeding events.

## Discussion

Thrombus formation on occluder device or in cardiac cavities is a relatively rare but severe complication relating to ASD closure. It can result in systemic embolism with detachment and activity of the thrombus both in the early and late follow-up if not promptly and effectively treated. Studies indicate that the incidence of ASD closure-related thrombosis is estimated 0.7% to 1% (11–16). Nevertheless, ASD closure-related thrombosis is still a serious adverse event which should not be neglected.

ASD closure-related thrombosis is more frequently seen in delayed than in early stage after the occluder release (5, 9, 12, 17, 18). However, thrombosis can occur during device implantation and any stage after occluder release (13, 19).

According to existing reports, the risk factors of ASD closure-related thrombosis are occluder device type (13, 20), atrial fibrillation, atrial septal aneurysm (13, 19), and prothrombin and/or platelet activation abnormality (21). Krumsdorf et al. (13) claimed that Amplatzer occluder was not associated with thrombosis in compare with other 7 types, based on retrospective analysis of 1,000 cases. Interestingly, the child of our case received an Amplatzer occluder and had no pre-existing risk factors. Fiarresga et al. (5) and Eren et al. (22) speculated that the thrombus was already formed in the delivery sheath when the left disc was first released. With the retraction of the occluder, a portion of the thrombus can attach to the left disc and thus be taken to the heart.

There is no current consensus regarding the therapeutic strategy for ASD closure-related thrombosis due to the rare occurrence. Antithrombotic agents are considered suitable options in both early and late stages of the thrombosis. Yorgun et al. (19) reported successful dissolution of early thrombus on an Amplatzer closure device using unfractionated heparin (UFH) and subsequent aspirin combined with warfarin. Willcoxson et al. (23) reported a case of acute ASD closure-related thrombosis in a 12-year-old boy receiving combined use of Abciximab and heparin for 12 h. With subsequent substitution with Aspirin and Clopidogrel, the child's thrombus completely dissolved in two weeks after the closure procedure.

Aside from antithrombotic therapeutics, to aspirate the thrombus by the transport catheter under close monitoring of TEE guidance (5, 22). If a more hazardous event is considered, such as large thrombus, device problem, or failure of medicinal intervention, surgery to remove the thrombus or the closure device should be prompted (24, 25).

Tirofiban is a reversible non-peptide platelet GPIIb/IIIa receptor antagonist that blocks fibrinogen binding to GPIIb/IIIa receptors to inhibit platelet aggregation and can prevent platelet thrombosis (26). In recent years, increasing evidence suggests tirofiban's role as a preferable option and recommendation for thromboembolic diseases, such as coronary heart disease and stroke, in various versions of guideline (27–30).

Limited number of reports indicated tirofiban's possible efficiency in thromboembolic complications relating to ASD closure in adults. Yazıłoğlu et al. (31) reported successful treatment with tirofiban for acute thrombus on the left disc in a patient with homozygous factor V Leiden mutation. The patient subsequently received lifelong warfarin therapy without recurrent thrombus. Vanderheyden M et al. (32) reported a patient with a 1-cm thrombus in both left and right atriums occurred 6 months after PFO closure. Interestingly, the thrombus was completely dissolved after 48 h of continuous intravenous infusion with recombinant tissue plasminogen activator and tirofiban, succeeding to failure by combined use of aspirin, clopidogrel and enoxistin for a week. Unfortunately, there is no report regarding tirofiban in treating ASD closure-related thrombosis in children.

In this case, the child achieved favorable outcome by combined use of tirofiban and heparin after experiencing acute thrombosis during ASD closure procedure. Our explanation for the outcome are: (1) the size of the thrombus was relatively small and we detected the thrombus immediately; (2) the activation of platelets was considered a pivotal mechanism underlying acute thrombosis (21), the combination of tirofiban and heparin was thought to induce stronger antithrombotic effect than use either of the two drugs.

Although the incidence of ASD closure-related is rare, the mobility and potential fragility of thrombus imply high risk of embolism. Therefore, effective and safe approaches to the management are required. In this case, our patient avoided rescuing surgical intervention and had a favorable outcome after treatment with combined use of tirofiban and heparin. We hope our experience will provide insights into the management of thrombosis relating to CHD interventional treatment.

## Data Availability

The original contributions presented in the study are included in the article/Supplementary Material, further inquiries can be directed to the corresponding author.
